# Lipoprotein lipase activity and mass, apolipoprotein C-II mass and polymorphisms of apolipoproteins E and A5 in subjects with prior acute hypertriglyceridaemic pancreatitis

**DOI:** 10.1186/1471-230X-9-46

**Published:** 2009-06-17

**Authors:** Inmaculada Coca-Prieto, Pedro Valdivielso, Gunilla Olivecrona, María José Ariza, José Rioja, Pilar Font-Ugalde, Carlota García-Arias, Pedro González-Santos

**Affiliations:** 1 Unidad de Lípidos, Servicio de Medicina Interna, Hospital Virgen de la Victoria, Málaga and Departamento de Medicina, Universidad de Málaga, Malaga, Spain; 2 Department of Medical Biosciences, Umeå University, Umeå, Sweden; 3 Laboratorio de Lípidos y Arteriosclerosis, Centro de Investigaciones Médico-Sanitarias, Universidad de Málaga, Malaga, Spain; 4 Departamento de Medicina, Facultad de Medicina, Universidad de Córdoba, Cordoba, Spain

## Abstract

**Background:**

Severe hypertriglyceridaemia due to chylomicronemia may trigger an acute pancreatitis. However, the basic underlying mechanism is usually not well understood. We decided to analyze some proteins involved in the catabolism of triglyceride-rich lipoproteins in patients with severe hypertriglyceridaemia.

**Methods:**

Twenty-four survivors of acute hypertriglyceridaemic pancreatitis (cases) and 31 patients with severe hypertriglyceridaemia (controls) were included. Clinical and anthropometrical data, chylomicronaemia, lipoprotein profile, postheparin lipoprotein lipase mass and activity, hepatic lipase activity, apolipoprotein C II and CIII mass, apo E and A5 polymorphisms were assessed.

**Results:**

Only five cases were found to have LPL mass and activity deficiency, all of them thin and having the first episode in childhood. No cases had apolipoprotein CII deficiency. No significant differences were found between the non-deficient LPL cases and the controls in terms of obesity, diabetes, alcohol consumption, drug therapy, gender distribution, evidence of fasting chylomicronaemia, lipid levels, LPL activity and mass, hepatic lipase activity, CII and CIII mass or apo E polymorphisms. However, the SNP S19W of apo A5 tended to be more prevalent in cases than controls (40% vs. 23%, NS).

**Conclusion:**

Primary defects in LPL and C-II are rare in survivors of acute hypertriglyceridaemic pancreatitis; lipase activity measurements should be restricted to those having their first episode during chilhood.

## Background

Among patients with acute pancreatitis, 1.3 to 3.5% are due to hypertriglyceridaemia, also known as hypertriglyceridaemic pancreatitis [1,2], some times relapsing and being even more severe than lithiasic acute pancreatitis [3]. Acute hypertriglyceridaemic pancreatitis forms part of the Chylomicronaemia Syndrome, defined as the presence of one or more of the typical signs (eruptive xanthomas, lipidaemia retinalis, recurrent abdominal pain or acute pancreatitis) in a patient with plasma triglyceride concentrations >22.58 mmol/L[4].

Genetic causes of the syndrome are rare and include deficiency of lipoprotein lipase (LPL), apolipoprotein C-II, and familial inhibitor of LPL. Other genes are also involved in the catabolism of chylomicrons, such as those for apolipoprotein E, apolipoprotein A-V [5] and glycosylphosphatidylinositol hgih density lipoprotein-binding protein [6,7] Patients with familial forms of hypertriglyceridaemia in combination with secondary acquired disorders (obesity, diabetes, pregnancy or drugs, including estrogens, retinoids, highly-active antiretroviral drugs) account for most individuals presenting with chylomicronaemia [8].

Although the presence of chylomicrons is necessary for the development of hypertriglyceridaemic pancreatitis and these are considered to be present when the triglycerides >11.29 mmol/L, it is common in out-patient clinics to see patients with much higher concentrations who are nevertheless asymptomatic and have not required admission for hypertriglyceridaemic pancreatitis. Few studies on the catabolism of triglyceride-rich lipoproteins have been published [9], with most reports concerning isolated cases with severe chylomicronaemia triggered by drugs or pregnancy [10-17]. Furthermore, additional interest concerns the identification of LPL-deficient patients for the potential use of intramuscular administration of an adeno-associated virus serotype 1 (AAV1) vector encoding the human LPL(S447X) variant cDNA (AAV1-LPL(S447X)), which normalized the chylomicronaemia in LPL-/- mice for more than 1 year [18] and is providing new data on humans[19].

We therefore studied LPL mass and activity, hepatic lipase activity, the levels of apolipoprotein C-II and apo E and apo A-V polymorphisms in order to detect any differences between persons with hypertriglyceridaemia who developed hypertriglyceridaemic pancreatitis and those who did not.

## Methods

### Patient selection

We studied 24 patients who were referred to our Lipids Unit after having hypertriglyceridaemic pancreatitis (HP Group). To be included in this group the patients had to have suffered at least one episode of acute pancreatitis (symptoms compatible with raised amylases in blood and urine and/or lipase in blood and morphological involvement of the pancreas on abdominal CT) and triglyceride concentrations on admission >11.29 mmol/L or, failing this, a lipaemic serum. All the patients with hypertriglyceridaemic pancreatitis were studied from several weeks to months after the episode of acute pancreatitis, when they were at home and with no symptoms.

In order to compare the different variables, a control group was recruited consisting of 31 patients referred to our Lipids Unit with severe hypertriglyceridaemia (triglycerides >11.29 mmol/L on at least one occasion), which thus represented a risk of having had hypertriglyceridaemic pancreatitis (HTG Group). As our centre has no paediatric unit, all the participants were older than 14 years of age.

All the patients and controls were being treated with diet and/or lipid lowering drugs. Due to ethical reasons, in no case was the medication stopped or changed because of inclusion in the study.

The clinical records of all the patients with hypertriglyceridaemic pancreatitis were reviewed retrospectively, in order to identify any factors predisposing to chylomicronaemia that could have led to the development of acute pancreatitis. Data were recorded on alcohol consumption, the presence of diabetes mellitus and its degree of control, dietary transgressions, pregnancy, and the intake of oestrogens or other drugs that could potentially increase triglyceride concentrations in predisposed patients (retinoic acid, antiretroviral therapy, corticoids, etc...). We considered moderate alcohol consumption less than 40 g in men and 20 g in women. No patients with alcoholic pancreatitis were included in our series. Previous diagnosis of co-morbidities, such as hypertension, vascular disease, fatty liver and coronary heart disease were also recorded. Because few relatives were available for lipid analyses, family history of hyperlipidemia relied mainly in patient's recall.

The study was approved by the Research and Ethics Committee of Virgen de la Victoria Hospital and all the patients gave written informed consent.

### Lipid and Lipoprotein Profile

Venous blood samples were obtained from each subject after a 12-hour fast. The baseline sample was used for lipid analysis, lipoprotein and apolipoprotein fractions and the second sample (drawn from the contralateral arm 15 minutes after administering sodium heparin (100 IU/kg) was used to measure the LPL lipase and hepatic lipase activity and the LPL mass.

The lipoproteins were separated by ultracentrifugation and later precipitation [20]. The separation of the chylomicrons in each sample was done by ultracentrifugation for 30 minutes at 105,000 × g. Because our technique is not able to distinguish between smaller chylomicrons and larger VLDL the lipoprotein obtained were chylomicron-like particles. After chylomicrons-like particles were removed, plasma was ultracentrifugated for 18 hours, 10°C, d 1.006 Kg/L at 105,000 × g, in order to separate VLDL. The infranatant fraction, containing LDL plus HDL, was reconstituted to the original volume and HDL was measured after precipitation of LDL [20]. Cholesterol and triglycerides were measured in plasma and in each lipoprotein fraction by commercial enzymatic methods (ABX, Montpelier, France). The plasma apolipoproteins (A-I, B-100, C-II, C-III, E] were quantified by commercial immunoturbidimetric methods (ABX, and DAIICHI, Tokyo, Japan). These measurements were all done at the Centro de Investigaciones Médico-Sanitarias (CIMES) of Malaga University, Spain.

### Lipoprotein Activity and Mass

The LPL activity assay was done on an Intralipid 10% emulsion and the hepatic lipase activity was measured using a gum Arabic-stabilized emulsion of triolein as previously published [21,22]. Each sample for LPL and hepatic lipase activities was assayed in triplicate and two standard samples were analysed in each assay. The activities of LPL and hepatic lipase are shown as mU/mL of plasma. One mU corresponds to nmol of fatty acid released per minute at 25° [23].

The LPL mass was measured by ELISA (19), using purified chicken antibodies to coat the wells and the monoclonal antibody 5D2, both against bovine LPL (Courtesy of Dr J. Brunzell, Seattle, USA). The mean values of all the samples were calculated for at least 3 different dilutions. The results are expressed in ng/mL. Purified bovine milk LPL was used as an assay standard.

Postheparin values from 20 healthy subjects were: LPL activity 56 ± 23 mU/mL (range 17–92) and LPL mass 254 ± 108 ng/mL (range 117–419).

### Polymorphisms of Apolipoproteins E and A-V

Genomic DNA was extracted by BioRobot^® ^EZ1 (QIAGEN). Genotyping for *Apo E *and *APO A5 *polymorphisms was carried out by PCR and restriction fragment analysis. Amplification reactions were performed in an iCycler iQ™ (BioRad) thermal cycler employing iQ™ Supermix (BioRad) as reaction mix. The primers and thermal protocols used were as previously described [24,25], with minor modifications.

### Statistical Analysis

Comparison of the quantitative variables between groups was done with the Student *t *test for independent data and analysis of variance (ANOVA), or the Mann-Whitney "U" test and the Kruskal-Wallis "H" test if the variables failed to adjust to normality. Analysis of the association of qualitative characteristics was done with the χ-square test or Fisher's exact test if the expected frequency was less than 5. All the tests were bilateral and the results considered significant if the p < 0.05. The database and the various tests mentioned were done with the programme SPSS 12.0 (SPSS Inc, Chicago, USA).

## Results

The 24 patients with hypertriglyceridaemic pancreatitis were mostly men and smokers, with a family history of dyslipidaemia, and half of them were moderate consumers of alcohol. The only difference between the two groups was a lower prevalence of overweight or obesity in the patients with hypertriglyceridaemic pancreatitis. About half of patients in each group were treated with fibrates. (Table 1).

**Table 1:** Clinical and demographic data

	HP N = 24	HTG N = 31	p
Age (years)	43 ± 11	46 ± 9	NS

Age first onset AP	40 ± 11	-	

AP Episo N°	2.3 ± 1.9 (1–9)		
1epis	9 (37.5%)		
2epis	9 (37.5%)		
≥ 3 epis	6 (25%)		

Sex (Men)	21 (90%)	28 (87%)	NS

Smokers	15 (62%)	19 (61%)	NS

Alcohol users	12 (50%)	17 (55%)	NS

AF	13 (54%)	13 (42%)	NS

BMI (kg/m^2^)	26.5 ± 4.0	29.3 ± 5.0	0.03
<25	10 (42%)	4(13%)	
25–30	10 (42%)	14 (45%)	
>30	4 (17%)	13 (42%)	

Diet only	11 (45%)	8 (26%)	
Fibrates	13 (54%)	18 (58%)	
Niacin	1 (4%)	0	NS
Fish oils	0	5 (16%)	
Statins	1 (4%)	4 (13%)	

Comorbidities			
Diabetes	7 (29)	9 (29)	NS
Hypertension	5 (21)	13 (42)	0.052
Fatty Liver	5 (21)	9 (29)	NS
Vascular disease	3 (12)	5 (16)	NS
Coronary artery disease	1 (4)	5 (16)	NS

AP: acute pancreatitis. AF: positive family history.

HP : hypertriglyceridaemic pancreatitis; HTG: severe hypertriglyceridaemia;

Over half the patients with hypertriglyceridaemic pancreatitis had consumed alcohol prior to the episode, four patients (20%) had diabetes mellitus which was to some degree poorly controlled, and one patient had just started steroid therapy for Evans Syndrome. No triggering factor was found in four patients. None of the patients with hypertriglyceridaemic pancreatitis was pregnant or had consumed estrogens. A positive family history of hyperlipidaemia was around 50% in both groups, but probably it was underestimated because many relatives could not be analyzed.

**Figure 1:**
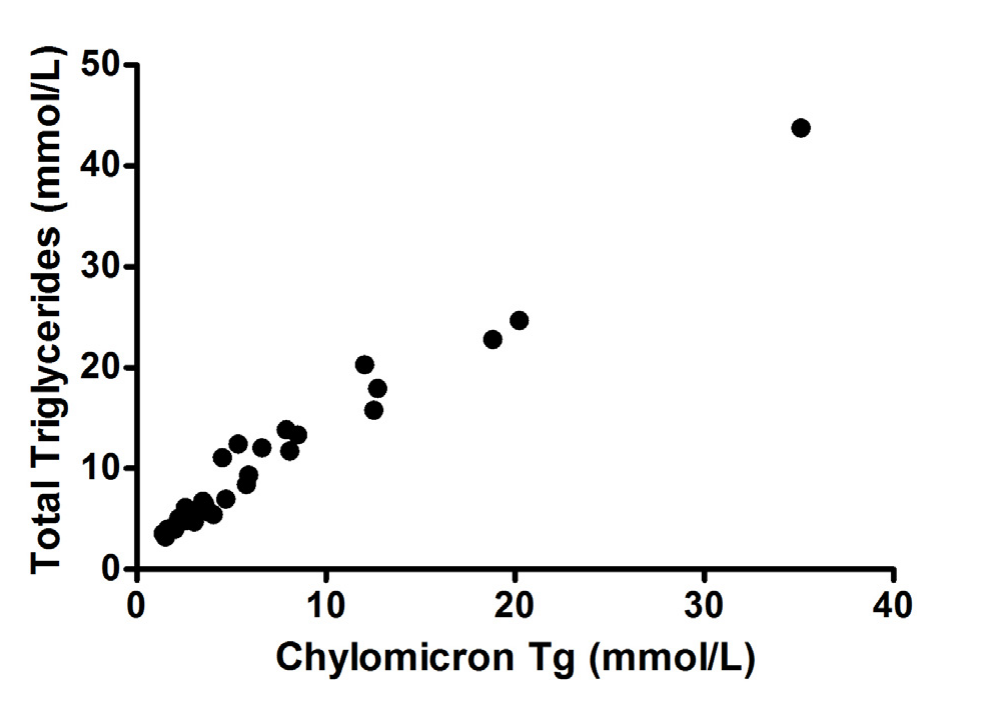
**Chylomicron-like triglycerides as a fraction of total plasma triglycerides**.

The group with hypertriglyceridaemic pancreatitis showed a non-significant tendency to have higher levels of triglycerides and cholesterol in the chylomicron-like fraction and a higher CIII/CII ratio. Fasting chylomicrons were present in 12/24 (50%) of the group with hypertriglyceridaemic pancreatitis (HP) and in 20/31 (64%) of the control HTG group without pancreatitis. Chylomicrons were even present in 12 patients whose fasting triglyceride concentrations were below 5.65 mmol/L (Figure 1). No patient was found to have a deficit of apolipoprotein C-II (Table 2).

**Table 2:** Serum lipids, lipoproteins, apolipoproteins and LPL mass and LPL and HL activities in postheparin and preheparin plasma samples

	HP N = 24	HTG N = 31	p
Total cholesterol	6.96 ± 3.72	6.56 ± 1.68	NS

TG	3.53 (2.07–8.75)	4.87 (2.70–8.41)	NS

VLDL-Chol	0.83 ± 0.44	0.91 ± 0.47	NS

HDL-Chol	0.91 ± 0.49	0.88 ± 0.39	NS

LDL-Chol	2.95 ± 1.47	3.28 ± 1.40	NS

VLDL-TG	1.39 (1.06–1.97)	1.41 (1.10–2.32)	NS

HDL-TG	0.34 (0.23–0.46)	0.31 (0.25–0.44)	NS

LDL-TG	0.58 (0.42–0.64)	0.53 (0.41–0.69)	NS

Chylomicron Chol	4.29 ± 5.43	2.17 ± 1.53	NS

Chylomicron TG	5.30 (2.73–11.52)	3.63 (2.30–6.40)	NS

Apo A-I	1.04 ± 0.36	1.10 ± 0.30	NS

Apo B-100	0.91 ± 0.32	0.96 ± 0.27	NS

Apo CII	0.11 ± 0.05	0.13 ± 0.07	NS

Apo CIII	0.17 ± 0.07	0.20 ± 0.09	NS

Apo E	0.09 ± 0.04	0.11 ± 0.06	NS

ApoCIII/ApoCII	2.8 ± 4	1.7 ± 0.7	NS

Apo E Genotype			NS
E3/E2	1 (4)	5 (16)	
E3/E3	19 (79)	22 (73)	
E3/E4	2 (8)	3 (10)	
E2/E4	2 (8)	0	

Apo A-V, allele S19W	8 (32)	7 (23)	NS

LPLactivity (mU/ml)	69 ± 44	92 ± 44	0.057

HL-activity (mU/ml)	164 ± 94	194 ± 84	NS

Posth-LPLmass (ng/ml)	312 ± 192	411 ± 195	0.06

Preh-LPLmass (ng/ml)	18 ± 11	22 ± 14	NS

Data shown as mean ± SD unless Tg shown as median (IQ range). Units in SI. Note: Chylomicrons were found only in 12 subjects in the group HP and in 21 of the group HTG. Apo E and apo AV genotypes mean number (%).

**Figure 2:**
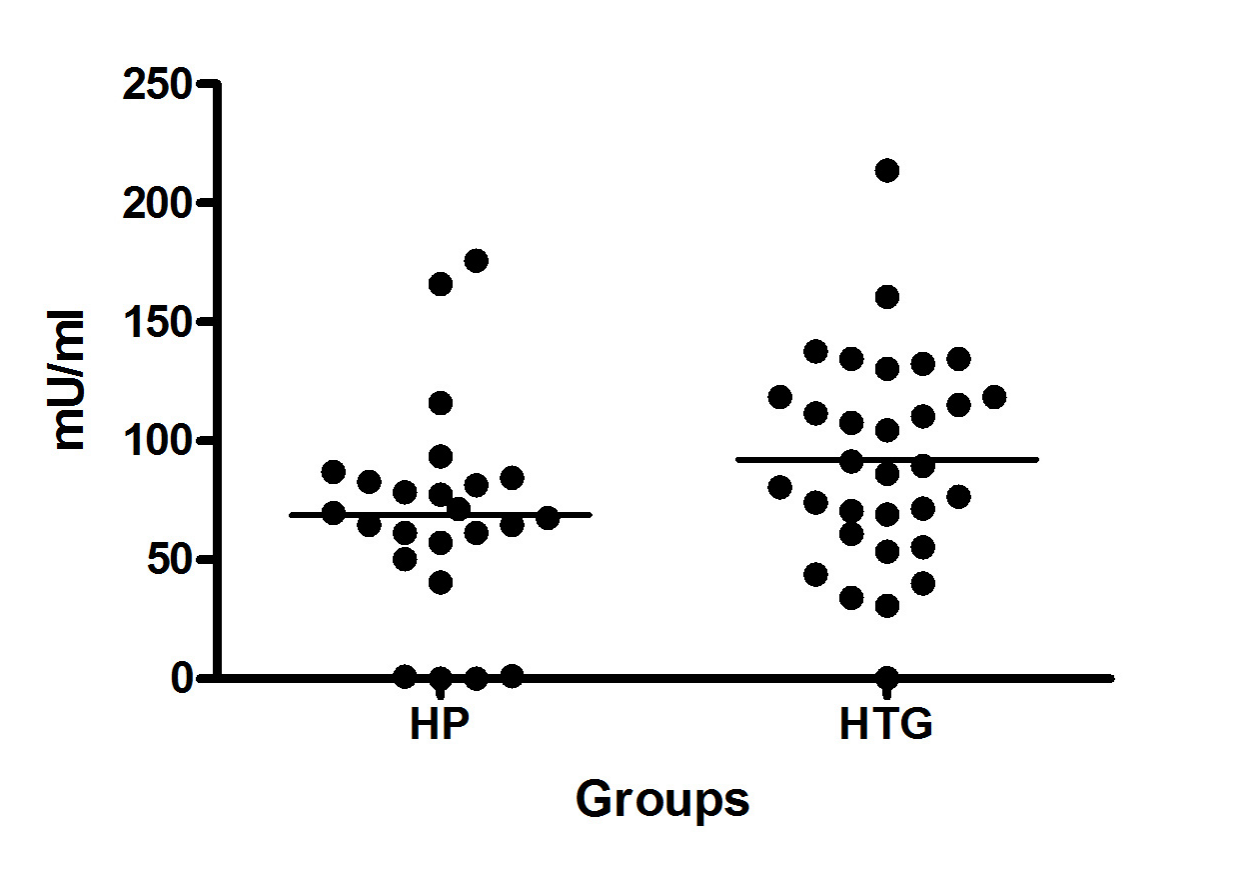
**Post-heparin LPL activity in both groups, identifiying 5 individuals with no activity**.

The patients with hypertriglyceridaemic pancreatitis had a lower post-heparin LPL mass and activity than the control group, but no differences were found in hepatic lipase activity, as expected (Table 2). Individual analysis of the post-heparin LPL activity (Figure 2) showed five persons whose LPL mass and activity was almost null. Four of these five patients with a deficiency came from the hypertriglyceridaemic pancreatitis group and one from the HTG group. The latter was the sister of a patient with a deficiency. Although she had never been admitted with acute pancreatitis she had suffered recurrent episodes of abdominal pain since she was a child. Accordingly, we decided to divide the group of hypertriglyceridaemic pancreatitis patients into two subgroups, one with a deficiency (HPD) and the other with no deficiency (HPND).

Table 3 clearly shows that the HPD patients were younger, had their first episode of hypertriglyceridaemic pancreatitis at an earlier age and a more recurrent course; they showed lower body-mass index than the patients with HPND and the HTG group. Moreover, the five patients with HPD had significantly higher levels of triglycerides, lipids in the fraction of chylomicrons-like and serum apo E, whereas they had significantly lower levels of LDL cholesterol and HDL cholesterol, as well as the serum concentration of apolipoproteins B-100 and A-1 (Table 4). (Table 4). By contrast, no differences were found in lipids, lipoproteins, apolipoproteins or post-heparin lipase mass or activity between the HTG and the HPND groups (Table 4). Indeed, no differences were found between the groups for apo E polymorphisms; although the rare S19W allele of the apolipoprotein A-V gene was non-significantly more frequent in the HPND (40%) patients than the HTG group (23%).

**Table 3:** clinical data of patients with LPL deficiency

	HPND N = 20	HTG N = 30	HPD N = 5	P
Age	47 ± 8	47 ± 7	23 ± 6	<0.001*

Age first onset AP	40 ± 11	-	6 ± 6	<0.001**

Episode AP number	1.75 ± 0.85	-	2.5 ± 0.6	NS

Sex (Male)	19 (95%)	27 (90%)	3 (60)%	NS

Smokers	15 (75%)	19 (63%)	-	0.006*

HLP AF	10 (50%)	12 (40%)	4 (80%)	NS

Fibrates	13 (68%)	18 (58%)	-	NS

BMI	27.4 ± 3.6	29.7 ± 4.8	21.3 ± 1.6	0.01

HPD: patients with LPL deficiency.

*Differences between HPD and the others groups.

**: Differences between HPND and HPD.

**Table 4:** Serum lipids, lipoproteins and apolipoproteins in the three groups

	HPND (N = 20)	HTG (N = 30)	HPD (N = 5)	P
TC	6.36 ± 2.53	6.75 ± 1.58	8.66 ± 6.86	NS

TG	2.67 (1.85–5.00)	4.81 (2.57–7.83)	13.32 (7.69–29.77)	0.006*

VLDL-Chol	0.78 ± 0.41	0.91 ± 0.49	0.98 ± 0.59	NS

HDL-Chol	1.01 ± 0.47	0.91 ± 0.39	0.39 ± 0.13	0.01*

LDL-Chol	3.21 ± 1.40	3.39 ± 1.32	1.45 ± 1.27	0.01 *

VLDL-TG	1.27 (1.02–1.81)	1.39 (1.07–2.49)	1.94 (1.45–3.22)	NS

HDL-TG	0.35 (0.28–0.46)	0.32 (0.25–0.46)	0.21 (0.14–0.44)	NS

LDL-TG	0.56 (0.39–0.64)	0.53 (0.43–0.69)	0.59 (0.46–1.00)	NS

Chylo-Chol	2.95 ± 3.34	2.22 ± 1.58	5.90 ± 7.55	NS

Chylo-TG	3.31 (2.52–7.40)	3.48 (2.21–6.60)	8.49 (5.2–23.81)	0.02**

Apo A-I	1.14 ± 0.29	1.11 ± 0.28	0.54 ± 0.17	<0.001 *

Apo B-100	0.98 ± 0.27	0.98 ± 0.25	0.56 ± 0.37	0.007 *

Apo CII	0.11 ± 0.06	0.13 ± 0.07	0.09 ± 0.05	NS

Apo CIII	0.17 ± 0.07	0.20 ± 0.09	0.19 ± 0.05	NS

Apo E	0.07 ± 0.04	0.11 ± 0.06	0.14 ± 0.03	0.005 *

CIII/CII	2 ± 3	1.7 ± 0.7	5 ± 7	NS

LPLactivity (mU/ml)	82 ± 34	95 ± 41	0.4 ± 0.5	<0.001 *

HL-activity (mU/ml)	179 ± 95	195 ± 86	107 ± 47	NS

Posth-LPLmass (ng/ml)	365 ± 164	422 ± 190	58 ± 27	<0.001*

Preh-LPLmass (ng/ml)	19 ± 10	22 ± 14	17 ± 12	NS

Data are shown as mean ± SD unless Tg shown as median (IQ range). HPD: patient with LPL deficiency. HPND: HP without LPL deficiency. TC: total cholesterol. TG: triglyceride. *: Differences between HPD and the others groups.** Differences between HPD and HTG group.

## Discussion

Our study focussed on the analysis of post-heparin lipoprotein-lipase activity and the level of apolipoprotein C-II in persons who had survived at least one episode of hypertriglyceridaemic pancreatitis in comparison with persons who were at risk for the same disorder due to having severe hypertriglyceridaemia with triglycerides >10 mmol/L [26]. The study led to the identification of five persons with a deficiency in LPL mass and activity, no case with a deficiency of apolipoprotein C-II and a borderline increase in apo A V S19W polymorphism in HP group.

Our findings are coherent with previous reports, in which the vast majority of cases with severe hypertriglyceridaemia (phenotype I or V) are due to the presence of one or more secondary causes. For example, just five of 123 patients with severe hypertriglyceridaemia (triglycerides>22.58 mmol/L) were deficient in LPL [27,28]. Additionally, no case of LPL deficiency was reported among 27 adults with hypertriglyceridaemic pancreatitis [29]. Moreover, among 129 patients with severe hypertriglyceridaemia referred to an Endocrinological Department for evaluation, including 26 with acute pancreatitis, no one was found to have LPL deficiency [9].

Our patients with hypertriglyceridaemic pancreatitis due to LPL deficiency were clinically different from the those with preserved LPL mass and activity, which explain their greater frequency of episodes of recurrent pancreatitis, the onset before adolescence, and the lower weight and body mass index than the patients with hypertriglyceridaemic pancreatitis and preserved LPL mass and activity. Analytically, besides the greater presence and levels of chylomicrons, the patients with a deficit had lower levels of LDL, HDL, apo B-100 and apo A-1. Moreover, the patients with hypertriglyceridaemic pancreatitis without LPL deficiency were more likely to have associations with important environmental factors, such as diabetes, obesity and alcohol consumption. The family association in some of these patients, the lipoprotein profile with marked hyperchylomicronaemia and the onset in childhood, all suggest a diagnosis of familial LPL deficiency, although a definitive diagnosis would require sequencing of the LPL gene or by measuring postheparin plasma LPL activity.

Our study found no differential clinical or analytical traits between the groups with hypertriglyceridaemic pancreatitis without LPL deficiency and the group of patients with severe hypertriglyceridaemia that could be used for identification of patients with hypertriglyceridaemia who might be predisposed to a pancreatic event. Notably, and as opposed to what might be expected [27], the amount of triglycerides and cholesterol in the chylomicrons did not differ between the two groups either.

The patients were analysed during the stable phase of their hyperlipidaemia, after several weeks or months of treatment and at least several months since their last episode of hypertriglyceridaemic pancreatitis. It is thus more than likely that if the group with hypertriglyceridaemic pancreatitis been studied during the acute phase of the disease and, not whilst they were stable, we would have obtained greater information on those environmental factors that could have triggered the disease, for example dietary transgression such as a high consumption of fatty food or alcohol, cessation of treatment or severe lack of diabetic control. By the contrary, the acute phase of pancreatitis is not the best clinical scenario to measure LPL activity (pain, nasogastric tube, prophilactic heparin, inflammatory state...).

Interaction of environmental factors with certain genetic polymorphisms may also have triggered the onset of hypertriglyceridaemic pancreatitis. The presence of the ε2 or ε4 allele in the apolipoprotein E gene has been associated with severe hypertriglyceridaemia [30], hypertriglyceridaemic pancreatitis during pregnancy [11] and higher postprandial levels of hyperlipidaemia [31]. Nevertheless, our groups did not differ in the frequency of the non apo E3/E3 genotypes. Certain polymorphisms in the apo A-V gene have been associated with triglyceride levels in a healthy population [32] and in persons with severe hypertriglyceridaemia [33,34] Indeed, severe chylomicronaemia with repeated hypertriglyceridaemic pancreatitis has been reported in two persons with apo A-V deficit [35,36] One mechanism proposed is that apo A-V lowers triglyceride concentrations by guiding VLDL and chylomicrons to proteoglycan-bound LPL for lipolysis [37] or simply acting as a cofactor of the enzyme. In a group of persons with hypertriglyceridaemia (triglycerides >3.80 mmol/L), the prevalence of the rare allele S19W of apolipoprotein A-V was found to be 19%, whereas it was only present in 4% of the control subjects [38]. The frequency of this rare allele in our patients was spread unequally; it was absent in the patients with LPL deficiency but was more common in the patients with HPND than in the controls with hypertriglyceridaemia, and may perhaps have contributed to a greater predisposition in the former for the development of pancreatitis due to the chylomicrons. Unfortunately, our study was underpowered to reach any conclusion.

Some limitations, however, should be taken into account interpreting our data. Patients were seen several months after the discharge of the bout of HP; second, many of them have been treated with fibrates, which not only modify lipids but also increase the LPL activity.

## Conclusion

We found that a primary defect in the catabolism of TG-rich lipoproteins was rare in patients who had survived a bout of hypertriglyceridaemic pancreatitis. Quantification of the LPL mass and activity should therefore be reserved for patients with a normal body mass index and recurrent acute pancreatitis that started in childhood or adolescence. None of the known factors, such as the presence and amount of fasting chylomicrons, the coexistence of accompanying disease such as obesity or diabetes, or the consumption of alcohol outside the acute phase, enabled the hypertriglyceridaemic patients to be differentiated from those with hypertriglyceridaemia who had developed hypertriglyceridaemic pancreatitis. Nevertheless, certain genetic factors, such as the 19W variant of apo A-V gene or other factors not studied here may predispose patients with hypertriglyceridaemia to manage postprandial fat worse and thereby make them more vulnerable to hypertriglyceridaemic pancreatitis. Further studies along similar lines and with a greater number of patients are required to confirm these findings.

## Competing interests

The authors declare that they have no competing interests.

## Authors' contributions

IC performed all clinical studies, recruited patients, and wrote the draft. PV conceived and designed the study, analyzed data and wrote the draft. GO conducted lipases measurement and made substantial contribution to the study. MJA performed genetic analyses. JR performed lipid and lipoprotein analyses. PFU performed statistical analyses. CGA recruited and looked after patients. PGS coordinated the study and made substantial intellectual contribution to conception and design. All authors read the draft and approved the final manuscript.

## Pre-publication history

The pre-publication history for this paper can be accessed here:

http://www.biomedcentral.com/1471-230X/9/46/prepub
